# Ableism in Applied Behavior Analysis: Historical Context of Services for Autistic People

**DOI:** 10.1007/s40617-025-01100-w

**Published:** 2025-11-23

**Authors:** Jennifer J. McComas, Claudia Drossel, Shawnna Sundberg, Mari-Luci Cerda, Susan Wilczynski, Hannah S. Beavis

**Affiliations:** 1 Department of Educational Psychology, University of Minnesota, Minneapolis, MN USA; 2 Eastern Michigan University, Ypsilanti, MI USA; 3 Lighthouse Learning Center, Lubbock, TX USA; 4 Ball State University, Muncie, IN USA

**Keywords:** Ableism, Behavior analysis, Autism spectrum disorder, Neurodiversity, Neurodivergent, Bias

## Abstract

Ableism is broadly defined as a set of behaviors and attitudes or, in behavior-analytic terms, as private events, deeply embedded within societal norms (Friedman & Owen, 2017). This paper explores the concept of ableism and its relation to applied behavior analytic services, with a particular focus on its implications for services provided to Autistic people. The purpose of the paper is neither to criticize nor to offer apologies for applied behavior analysis (ABA), but rather it is to aid the reader in understanding ableism by describing the context in which applied behavior analytic interventions were designed to attenuate some of the severe behavior that was distressing for the Autistic person and their caregivers (e.g., parents, staff). Examples of such behavior included self-injury, aggression, and extreme social withdrawal which were characteristic of individuals diagnosed as Autistic in the early days of ABA. The authors trace the evolution of ableism in ABA through to the present day, in which the heterogeneity in behavioral expression and needs of people diagnosed as Autistic are perhaps greater than ever, and some of those needs continue to go unaddressed. This paper, along with its companion article, “Ableism in Applied Behavior Analysis: A Beginner’s Guide to Understanding and Dismantling Ableism in Practice with Autistic People,” have incorporated both the limited available research regarding ableism in ABA-related services and lived experiences of some of the authors and other Autistic people whom the authors have provided services.

Friedman and Owen (2017) defined ableism as a set of attitudes (i.e., private events) and behaviors that function implicitly within societal norms, often without explicit awareness or recognition of biases against people with disabilities. Because ableism frequently involves private events, it is difficult to examine empirically, yet disabled people commonly experience ableist behaviors and attitudes (Friedman & Owen, 2017). Practitioners in the field of applied behavior analysis (ABA) are not exempt from engaging in ableist behavior (see Shyman, 2016). Therefore, this paper examines the history of ableism within ABA services for Autistic people, alongside the companion paper, “Ableism in Applied Behavior Analysis: A Beginner’s Guide to Understanding and Dismantling Ableism in Practice with Autistic People” (McComas et al., in press). The goal of the companion paper is to foster ongoing discourse on ableism in ABA and to offer practical behavior change strategies for beginning to dismantle it. The authors integrate and reference the limited available research on ableism in ABA-related services, first-person accounts of Autistic people, and the lived experiences of the authors and people they have served as practitioners.

## What Ableism Is and How it is Experienced

Ableism involves ideas, practices, institutions, and social relations that devalue and prejudge people with physical, intellectual, or psychiatric disabilities and rests on the assumption that disabled people are “less than” and need to be “fixed” (McClintock & Rauscher, 2007). Ableism is experienced as stereotyping, prejudice, discrimination, and social oppression by people with disabilities, who represent the largest marginalized group in the United States (Bogart & Dunn, 2019). Although approximately 1 in 4 adults in the United States (US) experiences some type of disability (Centers for Disease Control and Prevention, 2023), ableism is embedded in US culture (Friedman & Owen, 2017) and is not limited to the US (e.g., Leahy & Ferri, 2023). Ableism results in limiting beliefs about disability, influences how nondisabled people learn to interact with people with disabilities, and impacts why disabled people are often not included at the table for key decisions (Center for Disability Rights, n.d.). Ableism is a bias that presumes the norm of able-bodiedness and neuromajority^1^. As a result of ableism, disabled people regularly experience being largely invisible, disregarded, othered, pitied, distrusted/disliked, marginalized, and oppressed. Ableism, like other biases, often exists outside the person’s awareness (Amodio & Mendoza, 2010; De Houwer, 2019) and leads to assumptions about disabled persons’ character and quality of interpersonal relations, economic prospects, and limiting physical and social access to various life spaces. Accordingly, ableism manifests when non-disabled^2^ people diminish the perspectives of disabled people or neglect to prioritize their preferences, priorities, and concerns, thereby positioning the non-disabled person in a place to exert control over many significant aspects of disabled people’s lives. Like other “-isms,” ableism maintains the status quo in which members of a marginalized group have less power solely based on their membership in a group. In this context, power refers to the ability to influence decisions and control or autonomy over one’s life. Ableism can include shaping narratives (Tarvainen, 2019), access and control of resources, and participation in decision-making (Nario-Redmond et al., 2019; Stone, 1980). The perspective of disabled people being inferior to non-disabled people permeates language, written documents, and personal and professional interactions throughout society, including ABA, despite good intentions (benevolent ableism; Nario-Redmond et al., 2019).

### Why Ableism Exists: A Behavior Analytic Perspective

To understand ableism, it is essential to examine the various ways society exercises power over its members and influences perceptions of what is considered “desirable” and “undesirable.” As Keller and Schoenfeld (1950) stated,

[The culture] teaches the individual what [they] may and may not do, giving [them] norms and ranges of social behavior that are permissive or prescriptive or prohibitive. It teaches [them]…standards of … good and bad conduct. (p. 366)

Controlling agencies, such as governments and schools (Skinner, 1965), along with influential people, exert considerable influence over a culture’s contingencies (Skinner, 1981). Within the broader culture, social groups —composed of diverse populations—further shape, reinforce, suppress, or eliminate contingencies that sustain the group’s practices, leading to interlocking patterns of behavior (Skinner, 1957). These conditions give rise not just to isolated individual behaviors but to coordinated patterns of interaction among large groups of people (Tourinho & Vichi, 2012). As Skinner (1971) observed:

The practices of a culture, like the characteristics of a species, are carried by its members who transmit them to other members. In general, the greater the number of people who carry a species or culture, the greater its chances of survival…to the extent that it helps its members to get what they need and avoid what is dangerous, it helps them to survive and transmit culture. (p. 128)

Understanding how group contingencies shape human behavior is crucial to recognizing why biases persist, even when people adamantly deny holding them—especially when those biases operate outside their awareness. As members of a cultural group (e.g., behavior analysts), people tend to engage in behaviors that optimize access to reinforcement while minimizing the risk of punishment. For instance, behavior analysts may be more likely to reinforce verbal behaviors that reference topics such as data collection, behavioral functions, and contingencies.

Conversely, within the cultural group of behavior analysts, verbal behavior that includes mentalistic explanations of human behavior, intervention selection without consideration of controlling variables, or assessments of intervention effectiveness based on subjective impressions is more likely to be punished. Thus, members within the cultural group (i.e., “in group members”^3^) reinforce each other’s adherence to these cultural norms while punishing behavior that deviates from established group expectations. Additionally, out-group members (i.e., those who do not share the same culture) may be subjected to punishment—through practices such as social exclusion, dismissal of their views, or public criticism—while in-group members who participate in such punishment may receive reinforcement. Unfortunately, this dynamic can deepen divisions, fuel misunderstandings, and perpetuate misrepresentations, while decreasing the likelihood that in-group members will encounter evidence or perspectives capable of challenging their existing beliefs, including biased opinions about out-group members.

The interlocking behavioral contingencies involving multiple people, the verbal behavior reinforcing the assumptions underlying ableism, and the cultural consequences associated with hostile, ambivalent, and benevolent ableism together form the meta-contingencies that sustain and perpetuate ableism (Glenn, 2004; Saini & Vance, 2020). However, given that humans are consciously aware of only a small fraction of their own behavior (Skinner, 1957), it follows that many of the reinforcing meta-contingencies and individual contingencies that maintain ableist behavior often go unnoticed. We speculate that these might include:

- Social positive reinforcement by “doing good” for disabled people, regardless of whether we have considered the priorities of the disabled or Autistic person
- Social positive reinforcement in the form of social status afforded to neuro-majority people
- Tangible stimuli that are more readily accessible to neuro-majority people than to marginalized disabled people whose opportunities are limited by the current environmental arrangement
- Negative reinforcement (e.g., avoiding the discomfort that often accompanies an awareness of marginalization based on disability status)
- Verbal behavior and rule-governed behavior that assume neuro-majority as the norm (e.g., “I am successful because I work hard” as opposed to, “My success is due to both my hard work and the fact that the world is arranged to ensure a richer schedule of reinforcement and more opportunities because I am in the neuro-majority.”)

## Has Ableism Always Been a Problem in Applied Behavior Analysis? A Historical Analysis

Ableism extends far beyond the field of ABA and the autism community. Disability rights advocates have dedicated their lives and careers to challenging ableism and making significant sacrifices to drive legislative, policy, and systemic changes aimed at dismantling discrimination. In the US, early disability advocacy efforts date back to the 1800s with the establishment of formal education for Deaf and Deaf-Blind communities (Spaulding & Pratt, 2015), the invention of braille (Roth & Fee, 2011), and growing public outcry over the mistreatment of asylum patients (Lutes, 2002). By the 1960s and 1970s, disturbing revelations about the inhumane conditions in state institutions nationwide (Houston, 2020) galvanized the disability rights movement, coinciding with the emergence of ABA as a formal discipline. Leaders in the field of behavior analysis identified an opportunity to apply scientific principles to affect change for disabled residents. In 1964, Ogden Lindsley wrote:

Human behavior is a functional relationship between a person and a specific social or mechanical environment. If the behavior is deficient, we can alter either the individual or the environment to produce effective behavior. Most previous attempts to restore behavioral efficiency by retraining, punishment, or physiological treatment have focused on only one side of this relation: the deficient individual. This approach implies that normal people can function in all currently existing social environments, that deficient people can be normalized, and that there are no deficient environments. Scientists have only recently directly focused on the environmental side of deficient behavior functions and the design of specialized or prosthetic environments to restore competent performance (p. 41).

Lindsley’s focus on the environment influenced thinking about the effects of the environment on behavior, including Lawton and Nahemow’s theory of person-environment fit (1973), which persists in numerous fields today. Accordingly, Baer et al. (1968) kicked off the first issue of the *Journal of Applied Behavior Analysis* by shifting the field’s focus away from behavior reduction (Rohrer & Weiss, 2023) that characterized behavior modification practices at the time to the systematic analysis of the influence of environmental variables on socially meaningful behavior. Further, Goldiamond (1974) articulated a constructional approach to addressing problems by identifying the end goals and what skills are needed to achieve those goals (i.e., building behavioral repertoires) rather than identifying barriers and addressing those (i.e., eliminating undesired behavior) before working on skill-building (Abdel-Jalil et al., 2023; Scallan & Rosales-Ruiz, 2023).

In stark contrast to behavior analysts’ emphasis on modifying the environment to enhance socially meaningful behavioral repertoires, many approaches aimed at treating or “curing” autism rely on experimental and non-evidence-based interventions, including nutritional supplements, dietary restrictions, immunoglobulin and hormone therapies (such as secretin), chelation therapy, and other unproven methods (Lindgren & Doobay, 2011; National Autistic Society, n.d.; Schreck & Mazur, 2008; Schreck et al., 2016). Furthermore, even some evidence-based interventions may be viewed through ableist perspectives that frame Autistic people as needing to be “fixed” or made to conform to neuro-majority norms (e.g., response interruption/redirection), rather than being supported in ways that honor individual strengths and personal values. These efforts to eliminate the symptoms or “deficits” of autism often reflect an underlying ableist belief that there is something inherently “wrong” with Autistic people that must be corrected (Anderson, 2023; Botha & Cage, 2022; Bottema-Beutel et al., 2023; Kapp et al., 2013; Shyman, 2016). This is not to condemn Autistic people and the people who love them (e.g., parents, caregivers) for seeking desperately needed intervention to address severe behavior concerns such as self-injury, aggression, and disordered eating and sleep. These behavior concerns by themselves or as co-occurring conditions (Leader et al., 2021) take a significant toll on the Autistic person (Menezes & Mazurek, 2021) as well as the family unit (Meaden et al., 2010; Nealy et al., 2012; Sánchez Amate & Luque de la Rosa, 2024). Note it is the severe behavior problem(s), not the Autistic person, that requires intervention.

Although behavior analysis emphasizes systematic application of interventions to support socially meaningful behavior through the scientific principles of behavior analysis (Baer et al., 1968), we argue that behavior analysts have inadvertently played a significant role in perpetuating ableism against Autistic people. In many cases, ableist attitudes were evident in the emphasis on addressing particular forms of behavior (i.e., topographies) rather than environmental influences (i.e., function). This focus on behavior modification, rather than a functional-analytic approach, contradicts the principles outlined by Baer et al. (1968) and Goldiamond (1974). A key concern raised by Autistic people is the use of procedures designed to reduce stereotypic behavior (see Kapp et al., 2019). Notably, among the most egregious examples is behavior analysts’ use of contingent electric skin shock (CESS) to eliminate stereotypic behavior (see Msumba, 2021 p.87–93 for one account). According to first-person (Msumba, 2021) and witness accounts (Ahern & Rosenthal, 2010), CESS causes harm. To illustrate, *Life* magazine (1965) detailed Lovaas’s intervention in an article titled “Screams, Slaps, and Love.” In addition to slaps, electric shock was administered to an Autistic girl, Pamela, for drifting “off during [reading] lessons into her wild expressions and gesticulations” (p. 90) - essentially, for engaging in stereotypic behavior. When Pamela did not respond to “scolding and stern shaking,” (p. 90) she was described as “not having enough anxiety to be frightened” (p. 90) and was subsequently taken to a shock room. As described:

When she resumed her habit of staring at her hand…Lovaas sent a mild jolt of current…through the floor into her bare feet. It was harmless but uncomfortable. With instinctive cunning, Pamela sought to mollify Lovaas with hugs…But he insisted she go on with her reading lesson. She read for a while, then lapsed into a screaming fit. Lovaas yelling, “No!” turned on the current. Pamela jumped – learning a new respect for “No.” (p. 90)

The juxtaposition of this description in the *Life* magazine article and Baer et al.’s (1968) emphasis on socially meaningful changes can be difficult to reconcile. It would be easy to label Lovaas as a "bad person" while glorifying Baer, Wolf, and Risley as “good people.” However, such categorizations disregard the culturally mediated learning histories that shape and sustain biases and behavior. Moreover, perspective-taking is essential in recognizing biases and identifying the contingencies that must change to provide anti-ableist services. Although social validity of support for Autistic people is best assessed by Autistic people themselves (Chazin et al., 2024) and interested parties such as parents, teachers, and legal guardians (Wilczynski, 2024), it also reflects broader societal views (Baer et al., 1968). Consequently, behavior analysts—like other professionals—may be inclined to prioritize their perceptions of social validity over the perspectives of those most directly affected, perpetuating ableist approaches to goals, interventions, or outcomes of intervention.

For example, when other disciplinary professionals were largely ignoring and institutionalizing Autistic people, Lovaas stated (Lovaas, 1981) that he was creating socially meaningful improvements by increasing the individual’s ability to communicate, engage socially, and develop other skills that might allow the individual to leave those same institutions (p. ix). This is where it is useful, arguably essential, to hold two seemingly conflicting truths, allowing for a nuanced understanding of the complexity of the situation. Both of these are true: (a) Lovaas’ behavioral approach (e.g., Lovaas, 1981; Lovaas et al., 1973) resulted in improved communication and other habilitative skills as well as decreased self-injurious behavior that generalized and maintained for some participants, *and* (b) some of the methods, such as the use of arbitrary aversive stimuli to change behavior, are objectionable (see Wilkenfeld & McCarthy, 2020). From a historical perspective, Lovaas’ work represented an important step toward effective support for autistic individuals, given that other approaches, such as psychodynamic therapies (Lovaas et al., 1973), of the time had not resulted in meaningful improvements in skills or behavior (Brown, 1960). However, recognizing the societal context of that time does not justify the treatment of Autistic people. Since then, there have been multiple concerns raised about research and practice that followed (Arthur et al., 2023), and inhumane treatment (e.g., Cernius, 2022; Devita-Raeburn, 2016) in the name of applied behavior analysis. Moreover, as current professionals continue to publicly raise similar concerns (see Graber & Graber, 2023), there is an urgent need for the field of behavior analysis to critically examine both past and present practices. This reflection must prioritize the dignity, safety, and autonomy of Autistic people, ensuring that interventions respect their rights and well-being.

As others have noted (e.g., Lestremau Allen et al., 2024; Kirby et al., 2022), the field of ABA has made significant progress in improving the application of behavioral practices since the development of early interventions, such as those created in the 1970s and 1980s. This history serves as a critical reminder for behavior analysts to continuously question their assumptions about which goals to prioritize, what interventions are appropriate, how to encourage and prioritize self-determination (Anderson, 2023) and how socially and culturally meaningful the resulting changes truly are. By challenging these assumptions, we can take intentional steps toward practicing in anti-ableist ways.

## Evolution of ABA-Based Services for Autistic People as a Context for Ableism

The application of behavior-analytic principles to Autistic people with high support needs expanded further following Lovaas’s 1987 publication, which reported significant improvements in Autistic children who received intensive (i.e., more than 40 hours 1:1 treatment per week) ABA-based therapy. Foundational studies on ABA-based interventions provided empirical evidence supporting effective instructional procedures (e.g., Gaylord-Ross et al., 1984; Harris et al., 1990) and strategies for addressing severe behavioral concerns, such as aggression and self-injury, particularly in people with intellectual disabilities and significantly limited communication abilities (e.g., Carr & Durand, 1985; Wacker et al., 1990). Roane et al. (2016) highlighted the early use of ABA as an effective approach for addressing target behaviors of Autistic people and emphasized the importance of integrating behavioral interventions with developmental approaches to improve overall outcomes (Roane et al., 2016; Schreibman et al., 2015). ABA-based therapy resulted in demonstrable behavior change and began to be considered a *treatment for autism* (i.e., improving variables such as IQ; see Eldevik et al., 2009).

The diagnostic process is also relevant to ableism outside of and within the field of ABA. Although the term used by diagnosticians is *autism spectrum disorders*, we adopt instead the term *autism spectrum conditions* (ASC) in this article because the term disorder is often viewed as pathologizing and we seek not to perpetuate any discomfort Autistic people may have with the term. Articles written about the earliest behavior analytic interventions to enhance skill repertoires of Autistic children reflected the deficit model of autism common at the time. As Hewett (1965) stated:

The autistic child is a socialization failure. One of the major characteristics of autism which both illustrates and perpetuates this failure is defective speech development. (p. 927)

Although the article in which this quote appears described procedures that were effective for improving communication, through today’s lens, we recognize the ableist and deficit-focused attitude of this statement. Some of the earliest behavior analytic intervention research that addressed “self-destructive behavior” (e.g., Wolf et al., 1964) and communication deficits (e.g., Lovaas et al., 1966) was conducted during the time when “Infantile Autism” was the only diagnostic category in the American Psychiatric Association (APA) 3^rd^ edition (DSM-III, 1980) for autism; people with symptom onset after 30 months of age were instead diagnosed with “schizophrenia of early childhood” until DSM-III-R (APA, 1987). Further, Autistic people were generally characterized by impaired intelligence and communication. In contrast, those who did not have cognitive or language impairments tended to be diagnosed with Asperger’s syndrome (Mirkovic & Gérardin, 2019). Thus, the diagnostic category pertaining to autism as it was understood then was relatively homogenous in that most diagnosed people had to rely on substantial assistance to perform basic and instrumental activities of daily living. Practitioners learned to apply behavior principles to support the development of communication and other skills related to essential activities of daily living (da Silva et al., 2023) and academics (Watkins et al., 2022). Issues related to diagnoses have continued to influence ableism within the field of ABA over time. Since 2013, the diagnostic criteria for ASC, communication disorders, and intellectual disabilities have been distinct, meaning that an ASC diagnosis alone no longer provides specific information about an individual’s communication abilities or cognitive challenges and individuals diagnosed with ASC are more heterogenous than ever. This shift in diagnostic approach has led to an increasingly diverse autism population in terms of strengths and support needs. People with co-occurring communication disorders, intellectual disabilities, or both require significantly different interventions and supports compared to those with strong language and cognitive skills (García et al., 2020), yet all receive the same diagnosis of autism. Thus, the content of support is more relevant than the level of intensity (i.e., 40 or more hours per week; Lovaas, 1987) to individualized assessment and instruction.

The 1990s brought the emergence of center-based educational and early intervention programs for Autistic people, along with the formal establishment of the Behavior Analyst Certification Board (BACB), which standardized training and certification for behavior analysts. This period also marked the exponential growth of board-certified behavior analysts (BCBAs), further driving the widespread adoption of ABA-based services. However, the rapid increase in newly certified BCBAs has raised concerns about maintaining the quality of care, particularly as more professionals enter the field without sufficient oversight (Brown et al., 2023). Despite the rising number of newly certified BCBAs each year, the field continues to experience high turnover rates and persistent shortages of qualified professionals (Blackman et al., 2024; Mellon et al., 2023; Yingling et al., 2021). These shortages, coupled with limited supervision, can lead to time-saving shortcuts that compromise quality care (Morris, 2024a) and sideline the input of the people receiving services.

The 1990s also marked the beginning of substantial privatization across various sectors, including health and human services. This shift played a pivotal role in expanding ABA services for Autistic people. The development and growth of ABA-based services has been closely influenced by continuous shifts in insurance coverage. In the 2000s, several US states implemented mandates requiring insurance providers to cover autism-related therapies and treatments^4^. These mandates significantly increased access to behavior-analytic services (Berry et al., 2017), contributing to the transition of ABA-based services from an experimental approach to a widely accepted mainstream intervention (Hansel, 2013). As demand for ABA-based services grew alongside expanded insurance coverage, private equity (PE) firms took increasing interest in the field (Morris et al., 2024a). These firms began acquiring ABA service providers, which led to the corporatization of autism therapy services. For better or worse (Morris et al., 2024a; Smith & Lipsky, 1992), PE buyouts increased in the mid-2010s, driven by the potential for high returns from insurance reimbursements for ABA services (Batt et al., 2023). Recent estimates indicate that the ABA service industry is worth at least $4 billion (Batt et al., 2023; Morris et al., 2024a). The privatization and PE buyouts contribute to ableism due to the risk of prioritizing profit over service. Whereas nonprofit organizations are required to reinvest all profits they generate back into the organization in the form of staff development, training, and materials and services that their individual learners require and benefit from (see Morris et al., 2024a), for-profit organizations have the choice to direct profits to owners, shareholders, or both (see Heaslip, 2023; Morris, 2025). PE also takes over decision-making control of care management practices, despite having little or no expertise (Batt et al., 2023). With the influx of PE-owned ABA-based service provider organizations comes critical concerns regarding the quality of care for its recipients of these services, compromised ethical practice (see Garner, 2022), and geographic disparities in access to BCBAs (Yingling et al., 2021). Caution is warranted where (PE-managed) behavior analysts treat their Autistic clients as interchangeable (e.g., all requiring 40+ hours per week of therapy), as this reinforces an ableist perspective by reducing the individual to their diagnosis and making generalized assumptions based on disability rather than recognizing the person’s individuality and autonomy (Lombardo & Mandelli, 2022). Not all private and for-profit ABA-based service companies put profit before client interests or engage in more ableism than non-profit organizations. Nonetheless, we assert that all service providers, including but not limited to privatized and for-profit delivery of ABA-based services should be critically monitored for signs of objectifying clients as sources of income rather than as individual people who have strengths, preferences, priorities, and highly individualized service needs (see Batt et al., 2023; Morris, 2025). Overall, the rapid evolution and expansion of ABA-based services facilitated an increase in access to services for Autistic people. However, it also introduced the current challenges reported today with quality control and ethical service delivery (Garner et al., 2022).

## Summary

Behavior analysis training programs provide instruction on the philosophy, science, and application of behavior analysis; however, they may not fully address the complexities of supporting a highly heterogeneous population. To better prepare behavior analysts, training programs should incorporate interdisciplinary topics such as developmental psychology, genetics, chronic disease, verbal behavior, psychopharmacology, and general behavioral health principles, including sleep, nutrition, and mental health conditions (e.g., anxiety, depression) and their relationship to disability status (Cassidy et al., 2020; Cree et al., 2020). Each of these topics is relevant to the practice of ABA. For example, insufficient sleep is often a motivating operation that negatively impacts an Autistic person’s ability to benefit from high quality instructional practices. Similarly, targeting a behavior for change when it is not developmentally appropriate is not only likely to be ineffective but limits instructional time that can be dedicated to more essential skills. For example, one of the authors observed a registered behavioral technician trying to shape the accurate articulation of the word “three” in response to the discriminative stimulus, “How old are you?” because the behavior analyst who wrote the goal did not know three-year-olds often pronounce three as “tree” or “free.” Developing both specialized expertise and a broad skill set is essential for producing behavior analysts capable of effectively applying behavioral principles across diverse populations. However, we are aware that additional training in these areas would not and should not supplant the need for effective transdisciplinary and interprofessional collaboration (LaFrance et al., 2019; Slim & Reuter-Yuill, 2021).

Future research may reveal that ASC encompasses multiple distinct neurological conditions, each with unique etiologies, developmental trajectories, co-occurring medical conditions, and life expectancies (Monk et al., 2022). Although the field of ABA has demonstrated its potential in addressing behavior associated with these varying presentations, particularly when integrated with other professional disciplines, its application must remain responsive to each person’s unique needs. In other words, ABA must reaffirm its foundational emphasis on modifying environmental variables and delivering individualized, person-centered support (Lai et al., 2020; Schwartz & Baer, 1991) that addresses issues beyond skill acquisition such as quality of life (Schwartz & Kelly, 2021) and self-determination (Peterson et al., 2021). These practices should not only be evidence-based but also socially valid, ensuring they are meaningful and beneficial to those they serve. Rather than consider ABA as “effective for the “treatment of autism” (Association for Behavior Analysis International, n.d. [ABAI]), we encourage a reframing in terms of behavior analysis having contributed to effective instruction, behavior assessment, and intervention strategies designed to meet the highly individualized needs of Autistic people (Granpeesheh et al., 2009). This reframing would more accurately reflect the importance of valuing and supporting the unique strengths and identities of Autistic people while maintaining the application of the core principles of behavior analysis. Additionally, it would highlight the potential benefits of scientifically grounded, systematic support that fosters socially meaningful behaviors and expands individual repertoires while upholding autonomy, dignity, and well-being (BACB, 2020; Flowers & Dawes, 2023; Tincani et al., 2024). As Skinner expressed in a 1977 interview—an insight that resonates deeply with multiple authors of this paper—ABA should prioritize interventions that respect and enhance the autonomy, dignity, and lived experiences of people receiving support:

I want people to feel freer than ever before and feel more worthy of having achieved more than before. But these feelings are the by-products of the ways in which they are controlled. We don’t feel free when we are coerced; we do feel free when we are working for positive reinforcement, and I want to see lots of positive reinforcement. People will feel free and do things freely. Moreover, I want the contingencies in the social environment to be so good that they do much more than they do now, make more use of their talents than they do now.

The historical context of ableism in ABA, as discussed in this paper, has primarily centered on services for Autistic people based on reports that 82% of Board Certified Behavior Analysts focus on autism as their primary area of practice (BACB, 2025). Numerous systemic and structural factors have contributed to ableism within the field, and addressing these issues requires a concerted, ongoing effort. Encouragingly, meaningful progress is already underway in various aspects of ABA service provision (see Breaux & Smith, 2023; Chazin et al., 2024; Graber & Graber, 2023; Mathur et al., 2024; Taylor et al., 2019). Recent developments in dismantling ableism include a transition to focusing on compassionate care (Rodriguez et al., 2023; Taylor et al., 2019) and trauma informed care (Beaulieu et al., 2024) and a heavy focus on assent-based services (Morris et al., 2024b; Breaux & Smith, 2023). Despite a call to action for increased compassionate care in service delivery, work still remains on identifying the critical elements of compassionate care and how behavior analysts can begin to enhance training protocols that focus on learning key elements of compassionate care (e.g., empathetic listening, Taylor et al., 2019).

These topic areas have begun to emerge in rich discussions throughout the field, yet concerted effort is needed to further build methodological and practical impact within research and service delivery. Additionally, a call for greater collaboration and work within interprofessional teams will further progress the field toward meaningful impacts for individual outcomes (LaFrance et al., 2019; Slim & Reuter-Yuill, 2021). To support practitioners in actively challenging ableism, the companion paper, “Ableism in Applied Behavior Analysis: A Beginner’s Guide to Dismantling Ableism in Practice with Autistic People” (McComas et al., 2025), offers actionable steps and illustrative examples to promote more inclusive, person-centered approaches within ABA practice.

## Data Availability

No data were collected or analyzed for this paper, therefore there are no data to be made available.

## Footnotes

1 Neuromajority is used in place of “neurotypical” to refer to individuals whose brain, cognition, or neurological status/function is consistent with what is considered typical or dominant in a society (see Lestremau Allen et al., 2024).

2 It is important to note that people with disabilities can also exhibit ableism and internalized ableism (Kattari et al., 2018).

3 Those who are not like us are considered the “other” and are members of the “outgroup” (Kitzinger & Wilkinson, 1996). See Calma-Roddin (2024) for elaboration on in-group and out-group dynamics and teaching these concepts in psychology and education contexts.

4 “Therapies and treatments” is how the insurance industry refers to the supports that ABA-based providers offer. Given the medical nature of these terms, which invokes a medical model of disability, we prefer and suggest use of “supports” or “services” rather than “therapies and treatments.”
